# Multidisciplinary care for amyotrophic lateral sclerosis in rural Appalachia: Tales from the Clinic Coordinator

**DOI:** 10.1017/S1478951525101454

**Published:** 2026-01-08

**Authors:** Jennifer Zorotovich, Courtney Andrews

**Affiliations:** 1 Counseling and Human Services, East Tennessee State University, Johnson City, TN, USA; 2 Rehabilitative Sciences, East Tennessee State University, Johnson City, TN, USA

**Keywords:** amyotrophic lateral sclerosis (ALS), multidisciplinary care, rural health, clinic coordinator, coordinated care model

## Introduction

Amyotrophic lateral sclerosis (ALS) is a progressive neurodegenerative disease that primarily affects motor neurons, leading to muscle atrophy, respiratory failure, and ultimately death. Current treatment options focus on symptom management and maintaining quality of life rather than curing the disease (Hardiman et al. 2011). Multidisciplinary care teams are crucial in ALS management due to its complexity, requiring neurologists, various therapists, dietitians, respiratory specialists, and palliative care experts. Such care improves patients’ physical functioning, psychological well-being, and overall satisfaction (Hardiman et al. 2011; Sun et al. 2020).

In the Appalachian region of the United States, systemic and historical factors associated with rural healthcare create challenges for ALS patients in need of quality, specialized care. Rural residents often face barriers such as limited specialty providers, lower socioeconomic status (Kariisa and Seiber 2015; Zhang et al. 2023), transportation issues, and knowing that multidisciplinary, specialized care is available and where to find it. Neurological specialty care is particularly scarce, leading to delays in treatment for conditions like ALS (Rhudy et al. 2023). Free multidisciplinary care helps to bridge gaps between the patient and available healthcare. By alleviating financial strain, such services reduce burdens for families managing chronic illnesses such as ALS. Multidisciplinary teams also provide tailored health education and support, empowering families to manage ALS more effectively (Zhang et al. 2023). Care coordinators overseeing multidisciplinary clinics are pivotal in assisting patients in navigating through complex healthcare systems, ensuring that comprehensive treatment and follow-up are experienced in high quality ways that are critical for ALS symptom management (Sun et al. 2020). This paper is co-authored by the current Clinic Coordinator of East Tennessee State University’s (ETSU) Gary E. Shealy Memorial ALS Clinic as well as the Immediate Past Clinic Coordinator. Together, we share the historical backdrop of the clinic and provide insight into how multidisciplinary care is carried out in this setting. In doing so, we offer a model of care coordination that can be applied to a variety of healthcare settings where comprehensive, multidisciplinary care is offered, and we also provide recommendations to overcome common challenges faced by care coordinators.

## Gary E. Shealy Memorial ALS Clinic at ETSU

The Gary E. Shealy Memorial ALS Clinic was founded in February 2017 through the efforts of Dr. Faith Aiken, her late husband Gary E. Shealy, and ETSU. Recognizing the lack of ALS resources in Appalachia, they established a clinic to serve eastern Tennessee, Kentucky, North Carolina, and southwest Virginia. Patients in these areas are largely underserved and unserved considering severe barriers to care including transportation challenges, provider shortages, and overlapping healthcare costs. Rural residents consistently report poorer outcomes and less access to specialty care compared to urban populations (Pollack et al. 2023). With no multidisciplinary ALS clinic within 90 miles, our clinic provides essential access and is the only non-billing clinic in the state of Tennessee.

Our team includes professionals in respiratory therapy, physical therapy, speech-language pathology, occupational therapy, nursing, pharmacy, family medicine, behavioral health, assistive technology, nutrition, counseling, bereavement support, and end-of-life planning. ETSU-affiliated providers also manage faculty roles and as such are in a unique position to offer applied care experiences to train interprofessional student teams, combining patient-centered care with hands-on education.

The clinic began as a half-day monthly session at ETSU’s Internal Medicine Office. Growing demand led to relocation to the Nave Center for Rehabilitation and expansion into a full clinic day held monthly. Between July 2024 and July 2025, we provided 81 visits for 40 unique patients. Each visit involves the entire team, with patients remaining in 1 room while providers rotate through. Collaboration fosters continuity of care, which is essential in chronic disease management (McNeil et al. 2014). In rural areas, team-based care alleviates strain from geographic isolation and shortages while improving engagement and adherence (Reed et al. 2021). Moreover, interprofessional collaboration and transparent communication align with best practices, breaking down silos, and prioritizing patient needs (Parker et al. 2013; Martin et al. 2021). By adopting these strategies, the clinic strengthens patient flow, optimizes outcomes, and contributes to a resilient healthcare system (Bidwell and Copeland 2017; Sun et al. 2020).

## Role of the Clinic Coordinator

ETSU’s Gary E. Shealy Memorial ALS Clinic Coordinator ensures efficient delivery of care and oversees clinical operations. Responsibilities include managing electronic health care records, training and supervising a Graduate Assistant, managing a team of over 13 providers, overseeing workflow on clinic days, processing durable medical equipment (DME) orders and mental health referrals, monitoring intake, surveying patient experiences, and being the liaison between patients and providers on non-clinic days. Two groups of patients are seen, 5 in the morning from 9:00 to 12:00 and 5 in the afternoon from 1:00 to 4:00. On clinic day, a dry-erase board is used to track provider flow, reduce delays, and improve coordination. A table is drawn with patients listed on the y-axis and providers on the x-axis. Upon leaving to see a patient, the provider puts a dot and the time they started their appointment with the patient. The Clinic Coordinator tracks time spent with patients and checks in when 20 min have elapsed. This helps with workflow and manages the total amount of time patients are required to be physically present at clinic, which is important considering that those in progressed stages become easily fatigued. This model reflects “team-based care,” which emphasizes role clarity, shared responsibility, time management, and communication to improve outcomes. Effective communication among team members and with care-providing families is essential in offering quality care to patients. One way in which ETSU’s Clinic achieves this is by pairing providers to see patients together. For instance, physical and occupational therapists along with the assistive technology provider are grouped given the complementary nature of their disciplines, allowing for coordinated evaluations and discussions with patients and families. Similarly, speech language pathology and nutrition providers are paired as are the mental health counseling provider and the grief, bereavement, and end-of-life planning provider.

The Clinic Coordinator also manages logistical operations on clinic days such as space setup, attendance tracking, organizing lunch for providers, and snacks for patients. In addition to Clinic Coordinator roles, the current Clinic Coordinator also serves as the grief, bereavement, and end-of-life planning provider by offering emotional support, facilitating difficult conversations, and connection to resources to help patients and families prepare for end-of-life transitions. Such comprehensive coordination reflects best practices for clinics serving patients with complex conditions like ALS (Neel et al. 2024). The Immediate Past Clinic Coordinator simultaneously served as the speech-language pathologist provider to provide communication and swallow support.

Operational challenges often involve addressing unmet expectations from patients or staff. Specifically, many patients need assistance in navigating care outside of the clinic in ways that avoid insurance barriers, and many patients reflect not fully understanding the details associated insurance decisions surround coverage on various needs. The Clinic Coordinator facilitates problem-solving conversations to maintain a supportive environment and address barriers to care. Having a specialist such as a Nurse Case Manager or Medical Social Worker on these multidisciplinary teams is helpful in overcoming such barriers. Another common challenge for our patients is avoiding cost burdens on items that insurance does not cover. To overcome this, we often seek out and save information on available financial assistance programs either in the form of available funds for patients or through DME companies that offer programs to address larger co-pays. As mentioned earlier, both Clinic Coordinators managed their roles alongside being a provider. Both agree that operating simultaneously as Clinic Coordinator and provider presents time constraints and competing priorities that must be managed in strategic ways to provide quality services in both roles. This can be achieved through structured scheduling, utilizing time management tools, and implementing strong organizational techniques.

## Conclusion

ALS poses significant healthcare challenges, particularly in Appalachia, where access to specialty care is limited. Sustaining free multidisciplinary clinics is essential to improving patient outcomes and reducing disparities. Our workflow where patients remaining in 1 room while providers rotate illustrates an efficient, collaborative model of care. The Clinic Coordinator is central to managing providers, logistical operations, and patient needs, ensuring a patient-centered environment. This manuscript highlights the importance of integrated care strategies, collaborative practices, and resilient healthcare systems in addressing ALS, particularly in underserved rural populations.
